# Knoxville, Tenn.

**Published:** 1869-11

**Authors:** 


### Correspondence.

---

KNOXVILLE, TENN., Oct. 7, 1869.

DR. J. TAFT.—*Dear Sir:* In the REGISTER for September, under the head of "Another Experience," you give the results of an operation on a tooth of yours, of some nine years standing, etc. You say that on the removal of the plug some odor was perceived, which was "occasioned by the leakage through the imperfect border." Did you ever see a dead tooth which did not emit an odor? You may fill a dead tooth in the most perfect manner, and remove the plug in three months after the filling, and I will guarantee that you will find the odor not only in the tooth, but in the filling removed.

I am very glad that you have written this experience, as it will enable us to ventilate this subject a little. And let me start out by saying, that it is not so much the amount of gold which is put into a cavity, (and I would have all put in that can be got in) as the way it is put in. It is very evident that you have a large amount of gold put into that cavity, and, at the same time it is equally evident, that the gold was not perfectly packed against the walls of the cavity or it would not have leaked.

I hold (and I suppose you do the same) that if a cavity is perfectly filled it will stop the decay. If I did not firmly believe this, I would never fill another tooth. Would you?

I have seen many fillings as large as the one in your tooth, which have been in a much longer time without any leak, and yet, they have not so much gold in them as your's has, and now the question comes up. If your tooth was first filled with No. 4 or 6 (which is softer and more easily adapted to any surface than a higher number) and failed for want of perfect adaptation to the walls of the cavity, how will it turn out filled with No. 20? My opinion is, that if Dr. A has not much improved as an operator, his last filling will fail sooner than his first.

It is an easy matter to make hard fillings, but it is hard, very hard, to make them uniformly solid where they come in contact with the walls of the cavity.

Can this be done with high numbers of adhesive foils?

Can not No. 4 adhesive foil be made hard enough to resist mastication?

I used some Nos. 20 or 25 gold foil (soft) twenty-five years ago, (which I made myself by rolling it between copper plates) and it worked soft, for so thick a foil, but not soft enough for me to be assured that it was solid around the margins of the cavities. Some of those plugs are in yet, but they are hard looking customers. "Let us make haste slowly." Your friend,

J. FOUCHE.

---

EDITORS REGISTER:—I wish to say to the readers of the REGISTER that we are going to have the best meeting of the Ohio State Dental Society (which commences on the first Tuesday of December, next,) that we have ever had. This I say, not merely to get you to come to help make numbers, but that you may be able to avail yourself of the benefits of this meeting, for we are going to have a regular feast of good things, as all the good Dentists of our Association will be there, chuck full of such gladening information as will make us feel happy in our profession. Our worthy President, Dr. Horton, will be there to call us to order in his usual genial and happy way that will make it a pleasure for us to stop our greetings of each other after a year's separation, and give our attention.

Dr. Taft will be on hand with his heart overflowing with kindness toward all honest Dentists, and the greatest desire to give all who aspire to high rank in their profession, that knowledge which he is always ready to impart in the most instructive manner. We hope also to have with us once more our old and and much honored chemical friend, Dr. Watt, who will tell us just exactly what chemical effect may be expected by some new agent that may be introduced into Dental practice by Dr. Spellman. Dr. Palmer will have with him some of the best pointed pluggers now in America, in which he has developed his ideas of perfect pluggers. And Dr. Harroun will tell any who desire to know how the dumb may be made to speak. Dr. Blount is expected to be ready with a short dissertation upon office etiquette, which will be very pleasant when delivered with his usual gentle manner and smiling face. Dr. Keely will tell us about fine plugs. Dr Herriot asking him questions, which will be expected to enthuse him upon the subject.

But we have not began to tell you what you will hear from Drs. Rehwinkle, Berry, Smith, Buffett, Butler, and a score of others who will be with us. But I will only say farther, *come*, and my word for it you will go home pleased and benefited, feeling more happy for your existence and better satisfied with your profession. W. M. H.

